# The role of ribosomal DNA methylation in embryonic development, aging and diseases

**DOI:** 10.1186/s13072-024-00548-4

**Published:** 2024-07-31

**Authors:** Fei Yang, Xutong Guo, Yiming Bao, Rujiao Li

**Affiliations:** 1 Present Address: National Genomics Data Center, China National Center for Bioinformation, Beijing, 100101 China; 2 Beijing Institute of Genomics, Chinese Academy of Sciences, Beijing, 100101 China; 3 University of Chinese Academy of Sciences, Beijing, 100049 China

**Keywords:** rDNA, Methylation, Aging, Embryonic development, Disease

## Abstract

The ribosomal DNA (rDNA) constitutes a remarkably conserved DNA sequence within species, located in the area of the nucleolus, and responsible for coding three major types of rRNAs (18S, 5.8S and 28S). While historical investigations into rDNA focused on its structure and coding capabilities, recent research has turned to explore its functional roles in various biological processes. In this review, we summarize the main findings of rDNA methylation with embryonic development, aging and diseases in multiple species, including epigenetic alterations, related biological processes and potential applications of rDNA methylation. We present an overview of current related research and identify gaps in this field.

## Introduction

**Fig. 1:**
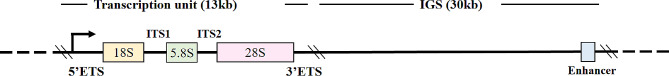
The structure of rDNA in human cells

Ribosomal DNA sequences are essential components of all eukaryotic genome sequences. In humans, the main rDNA unit spans approximately 43 kb, comprising a 13 kb transcribed region containing sequences encoding the 18S, 5.8S and 28S rRNAs, along with a 30 kb intergenic spacer (IGS) [1] (Fig. 1). Additionally, rDNA comprises a separate cluster of approximately 2.2 kb repeated genes that form the 5S rRNA [2, 3]. The critical components of IGS encompass upstream control elements (UCE) and core promoter elements (CPE), as well as other regulatory regions. rDNA is the only DNA present within the nucleolar volume [4], arranged in tandem repeat clusters in the nucleolar organizer regions (NORs), on the short arms of acrocentric chromosomes. rDNA gene sequences are highly conserved within species, characterized by high repeatability, high transcription and large amounts of methylation, which lead to the loss of gene expression under most of circumstances [5, 6]. In humans, rDNA sequence is predominantly located on chromosomes 13, 14, 15, 21 and 22 [7]. The region encoding 5S rRNA is primarily situated on chromosome 1 [8]. In mice, these sequences are found on chromosomes 12, 15, 17, 18 and 19 [9, 10], with the region encoding 5S rRNA mainly located on chromosome 8 [11]. Human and mouse sequences encoding rRNAs are highly conserved (percent identity: 5S = 100%, 5.8S = 99%, 18S = 99%, 28S = 85%) [12]. The primary function of rDNA is to form ribosome subunits. rDNA is first transcribed into rRNA in the nucleus by RNA polymerase I (pol I), a rate-limiting factor in ribosomal biogenesis. Additionally, some cell signaling pathways such as MAPK mTOR, MYC, PKC, p53 and RAS/ERK pathways also influence the transcription process. Subsequently, rRNA is processed and modified to assemble with the ribosome protein into the final ribosome subunit. Following ribosomal subunit synthesis, it began to play more prominent roles, such as regulating protein synthesis and transcription factor activity, participating in some post-translational modifications and regulating cell cycle [13, 14]. To meet the different demands of protein synthesis in various tissues under different conditions, multiple modalities such as epigenetic regulation are served to trigger the activation or silencing of rDNA transcription [15–17].

DNA methylation usually refers to a modification formed by the addition of methyl groups to the 5’C position of cytosine by DNA methyltransferase (DNMT) [18, 19]. Serving as an essential epigenetic regulator, it plays roles in various biological processes. Relevant research and data have been integrated into epigenetic databases to facilitate researchers’ understanding of advancements [20–22]. rDNA, as one of the key regions of methylation in the human genome, has at least 1500 CpGs on its transcription unit [23]. The methylation levels of rDNA vary across different tissues. A study reported the methylation levels of rDNA promoters in various tissues of adult mice as the following: liver 34%, spleen 53%, brain 48%, testis 25%, and ovary 30% [24]. As far back to 2001, study in mice had clearly identified that methylated rDNA copies are transcriptionally silent [17]. Methylation-mediated silencing of rDNA transcription can contribute to various biological processes such as organismal development, aging, and disease occurrence.

However, due to the high repeatability, near-mitochondrial characteristics and substantial size of rDNA, a lot of relevant information was lost during analysis of next-generation sequencing data, leading to the exclusion of rDNA sequence from the reference genome for a long time. Given this circumstance, research focusing on rDNA methylation is relatively constrained, with even fewer comprehensive reviews on the subject [5, 25]. In this review, we comprehensively summarize the current status and potential applications of research on the relationship between rDNA methylation and embryonic development, aging, and diseases across different species and tissues (Table 1). Additionally, we will provide insights into future directions for rDNA research.

**Table 1:** Correlation between rDNA methylation and embryonic development, aging, and diseases

Trait type		Trait	Tissue	Species	rDNA region	Methylation state changes	Other related changes	Reference
Development		Embryonic development	Sperm, germinal vesicle oocytes, metaphase II oocytes, zygote and blastocysts	Mouse	Promoter	Hypermethylation compared to adult tissues		[24]
			Sperm	Mouse	Promoter, transcribed region and IGS	Hypermethylation in aged sperm	(Peri)Centromeric minor and major satellite DNA, and interspersed LINE1 T repeats hypermethylation in aged sperm	[26]
				Rat	Transcribed region	Hypermethylation in aged sperm	-	[27]
				Bull	Transcribed region	Hypermethylation in aged sperm	-	[26]
				Marmoset	Transcribed region	Slight hypermethylation in aged sperm	-	[26]
				Human	Promoter and transcribed region	Hypermethylation in aged sperm	-	[26]
			Oocyte	Mouse	Promoter and transcribed region	Hypermethylation in aged oocytes	-	[28]
				Human	Promoter	Hypermethylation in oocytes from aging ovarian	Methylation changes between individual oocytes from the same donor are less than 5% in most samples	[28]
				Zebrafish	Transcribed region	Hypermethylation	Germline amplification	[29]
			Embryonic day 4.5	Mouse	Promoter	Embryo methylation lower than extra-embryo	-	[24]
			Gonads and Liver cells of embryonic day 13.5 and 18.5	Mouse	Promoter	Hypermethylation compared to primordial germ cells	-	[24]
			Liver cells of embryonic day 18.5	Mouse	Promoter	Hypermethylation compared to embryonic day 13.5	-	[24]
Aging		Aging	Blood	Rat	Promoter	Hypermethylation	-	[14]
				Human	Promoter	No significant change	-	[14]
			Bone marrow cells	Mouse	Promoter	Hypermethylation	The number of rDNA copies increased and the levels of pre-rRNA transcripts decreased	[30]
			Fibroblasts	Human	Promoter	Hypermethylation	-	[31]
			Skeleton muscle	Mouse	Promoter	15 sites were hypomethylated and 345 sites were hypermethylated	-	[32]
			Heart	Rat	Promoter	Hypermethylation	-	[14]
			Kidney	Rat	Promoter	Hypermethylation	-	[14]
			Liver	Rat	Promoter	Hypermethylation	-	[14]
					Transcribed region	Hypermethylation	-	[27]
					Promoter and transcribed region	Hypermethylation	-	[33]
				Human	Promoter and transcribed region	Hypermethylation	-	[33]
Diseases	Neurological diseases	Alzheimer’s disease (AD)	Cerebral cortex	Human	Promoter	Hypermethylation	An increase in overall rDNA content	[34]
		Mild cognitive impairment (MCI)	Cerebral cortex	Human	Promoter	Hypermethylation	An increase in overall rDNA content	[34]
		Schizophrenia (SCZ)	Neuronal and oligodendrocytes	Human	Transcribed region	No significant change	No significant change in rDNA copy number	[35]
		Autism spectrum disorders (ASD)	Brain	Human	Transcribed region	No significant change	No significant change in rDNA copy number	[35]
	Hematologic diseases	Myelodysplastic syndrome (MDS)	CD34 + cells	Human	Promoter	Hypermethylation	Decreased expression of rRNA, and alternation of ribosomal biogenesis	[36]
	Genetic disorders	Werner’s syndrome (WS)	Fibroblasts	Human	Promoter, transcribed region and IGS	Hypermethylation	The steady-state levels of 28S rRNA remained constant over the life span of both normal and WS fibroblasts	[37]
		Down syndrome (DS)	Whole blood	Human	Promoter	Hypermethylation	The number of rDNA copies increased	[38]
	Gender-specific cancers	Breast cancer	Breast	Human	Transcribed region	Hypermethylation	Increased methylation is associated with inhibition of estrogen receptor expression	[39]
			Breast	Human	Promoter and transcribed region	Hypermethylation	Lower levels of rDNA methylation demonstrated significantly poorer rates of disease-free survival and overall survival	[40]
		Ovarian cancer	Ovary	Human	Transcribed region	Hypermethylation	Overexpression	[41]
		Cervical cancer	Cervical intraepithelial neoplasia tissue	Human	Promoter	Hypomethylation	Decondensation of rDNA, and rRNA overexpression	[42]
		Endometrial carcinoma	Endometrium	Human	Transcribed region	Hypermethylation	Morphological changes in the nucleoli, ac-companied by overexpression of rRNA	[43]
		Prostate cancer	Prostate	Human	Transcribed region	No significant change	rRNA overexpression	[44]
			Prostate	Human	IGS	Hypomethylation	Increased variation	[45]
	Other types of cancers	Oral squamous cell carcinoma (OSCC)	Oral	Human	Promoter and transcribed region	No significant change	Decreased expression of rRNA	[46]
		Esophageal cancer	Esophagus	Human	IGS	Hypomethylation	Increased variation	[45]
		Hepatocellular carcinoma	Liver, cfDNA	Human	IGS	Hypomethylation	Increased variation	[45]
		Colorectal cancer	Colorectum, cfDNA	Human	IGS	Partial tumor samples show hypomethylation	-	[45]
		Lung cancer	Lung, cfDNA	Human	IGS	Partial tumor samples show hypomethylation	-	[45]

## rDNA methylation and embryonic development

It is widely recognized that during mammalian embryonic development, DNA methylation undergoes dynamic changes, including significant genome-wide “erasure-reconstruction” processes, with the exception of parental imprinting [47–49]. However, research examining the correlation between rDNA methylation and embryonic development stage is notably deficient in systematic approach. It predominantly concentrates on isolated developmental processes in mice, supplemented by studies concerning reproductive cells across various species.

The formation and maturation of germ cells are prerequisites for embryonic development. A study has reported that the variation in rDNA methylation among oocytes derived from the same individual in the majority of humans is less than 5% [28]. In a study conducted on mice, the promoter regions of rDNA in sperm (5.9%), germinal vesicle oocytes (5.4%), metaphase II oocytes (3.2%), zygotes (2.6%) and blastocysts (1.9%) exhibited significantly lower methylated levels compared to those of adult tissues, such as testis (25%), ovary (30%), liver (34%), brain (48%), spleen (53%) [24]. Several studies have identified an increase in rDNA methylation in germ cells with advancing age across various species. It has been discovered that in human sperm, the methylation levels of rDNA transcription units (including UCE, core promoter, 18S rDNA, and 28S rDNA) increase with age, similar to α-satellite DNA and LINE1. Similarly, methylation levels of rDNA in mouse sperm (including intergenic spacer, gene promoter, 18S, and 28S) also increase with age. Additionally, in bovine and marmoset sperm, methylation levels of rDNA (18S and 28S rDNA) rise with individual age, with marmosets showing a slight increase in magnitude [26]. Subsequent research suggested that human ovarian aging is associated with increasing oocyte methylation of the rDNA promoter and UCE, while a weak correlation of oocyte methylation with rDNA promoter, UCE, and 28S rDNA was found in mice [28].

After the mouse zygote undergoes cleavage to form a blastocyst, between embryonic day 4.5 (E4.5) and embryonic day 5 (E5), the blastocyst begins to implant into the uterine wall. After implantation, de novo methylation of rDNA promoters occurs, with the overall methylation level in the embryo (13.9%) being lower than that in the extra-embryo (31%) at embryonic day 7.5 (E7.5). Based on this, the rDNA methylation levels of gonads and fetal liver cells were examined at embryonic day 13.5 (E13.5) and embryonic day 18.5 (E18.5), revealing that the levels were higher than those of primordial germ cells at the respective time points. Additionally, liver rDNA promoter methylation increased from 16.3% at E13.5 to 22.2% at E18.5. These results suggest that de novo methylation of the rDNA promoter is inhibited during germ cell development and global methylation occurs only after implantation. It was also speculated that nucleosome remodeling and histone deacetylase (NuRD) might play a role in the maintenance of hypomethylation of rDNA. Furthermore, during the period from conception to weaning, research has shown a linear correlation between growth limitations resulting from protein restriction and alterations in DNA methylation of rDNA in mouse offspring, which persists into adulthood [50]. These findings offer new insights and perspectives for the study of the methylation characteristics of rDNA during development [24]. Some researches have shown that ribosome biogenesis plays a crucial role in maintaining the fate determination of embryonic stem cells and developmental homeostasis of mammalian [51, 52]. We speculate that some pivotal modifications in rDNA methylation during embryonic development may regulate rDNA transcription and silencing, thereby tightly controlling ribosome biogenesis and promoting the progression of embryonic development at appropriate stages. The Lower levels of rDNA methylation observed at the zygote and blastocyst stages may allow for extensive rDNA transcription, facilitating abundant synthesis of ribosomal RNA essential for the continued differentiation and development of embryonic cells. Therefore, we contend that studying rDNA methylation in embryonic development will contribute to a deeper understanding of the molecular mechanisms of embryonic development and have potential implications for the understanding of the pathogenesis of developmental diseases and applications in embryonic stem cell research.

Apart from mammals, the rDNA of the model organism zebrafish has also been studied during development. Research has shown that zebrafish maintain global methylation levels during sex differentiation without any stage similar to genome-wide methylation erasure in mammals. A specific type of rDNA located in the major sex-linked locus in oocytes (referred to as fem-rDNA) has been identified. Fem-rDNA amplification and demethylation during early development of zebrafish suggest that fem-rDNA amplification is implicated in sex determination, helping to explain the non-Mendelian sex ratio in zebrafish [29].

## rDNA methylation and aging

rDNA methylation can be considered a more effective biomarker of biological aging compared to other genomic components due to its comparable accuracy, more concentrated sites, and evolutionarily conserved characteristics [23, 53]. The theory proposed in 2008 suggested that the instability of the rDNA triggers aging and shortens individuals’ life span, which is called “rDNA theory of aging” [53, 54]. Many studies have explored the relationship between rDNA methylation and aging across different species and tissues, with the majority of findings indicating site-specific or overall hypermethylation of rDNA triggered by aging. In blood, elevated rDNA methylation and decreased rRNA levels were reported later in life in rats, although a comparable increase was not conclusively observed in humans [14]. The author further conducted a 9-year longitudinal study on the quality of life in individuals aged 60 to 89 years, revealing a negative correlation between rDNA methylation levels and cognitive abilities, as well as survival chances in the elderly population [14]. In bone marrow, it was observed that with increasing age, there is an elevation in the methylation level and copy number of rDNA, accompanied by a decrease in the levels of pre-rRNA transcripts using 2-year-old and 4-week-old mice [30]. For human fibroblasts, a 2018 report demonstrated an increase in rDNA methylation during in vitro aging [31]. For skeleton muscle, 345 CpG sites in rDNA were found hypermethylated in aged muscle compared to young muscle of mouse, while the number of hypomethylated CpG sites was only 15 [32]. In the heart and kidney of rats, with aging, the methylation of some CpG units in the promoter region increases [14]. For the liver, several studies have reported an age-related increase in DNA methylation at multiple regions of rDNA [14, 27, 33]. Methylation levels of three regions of the rDNA gene locus (promoter, 5’ of 18S, and 5’ of 28S sequences) were measured in young (two months), adult (10 months), and old (18 months) rats, revealing an age-associated increase in DNA methylation [33]. Additionally, a similar increase in methylation occurring in human liver cells was documented for the first time [33].

Based on the age-related changes in rDNA methylation described above, several age prediction models have been developed, utilizing the conservative characteristics of rDNA methylation to assist in the study of aging process. One model has been developed to predict individual’s age based on 15 CpGs within the rDNA transcription unit of human sperm. This model utilizes a relatively small amount of sample size of approximately10 sperm cells, with a prediction error of less than 3 years [26]. Furthermore, the effectiveness of rDNA methylation sites as a clock marker to predict the age of an individual has been tested across multiple distant species, including humans, mice, and dogs. The performance of rDNA is significantly better than that of clocks constructed with other genomic CpG sites as markers, and it is considered to be a reliable predictor of age due to its conserved performance across different species [23]. Among approximately 13,000 base pairs of rDNA sequence in mice, researchers identified 620 CpG sites (66.8%) that provide information about age. Interestingly, many of these CpG sites are conserved across distantly related species; for instance, over 70% of human CpG sites in the 18S and 5.8S genes of rDNA can be found in species as diverse as zebrafish. The elastic network regression model, constructed using methylation levels of 46 rDNA loci, has achieved accurate and precise estimation of the age of individual European lobsters, which will hopefully have economic and ecological value for fisheries management [55]. Furthermore, combining the rDNA methylation clock with genome-wide methylation clocks identified across the entire genome could potentially enhance the accuracy of age prediction [56–59].

Various hypotheses have been proposed regarding the potential cell biological mechanisms through which rDNA methylation may be involved in aging, including nucleolar stress, regulation of RNA polymerase affecting transcription rate, among others. A replication fork barrier (RFB) has been identified within human 47S rDNA, with essential elements including the Sal box and transcription termination factor-1 (TTF-1). Interestingly, it is active only in the hypomethylated rDNA copy, and its mechanism of action is to cooperate with TTF-1 to terminate RNA pol I’s transcriptional activity. The different methylation status of rDNA copies, therefore, could lead to genomic instability, potentially contributing to the development of cancer [60]. Besides, impaired ribosomal biosynthesis due to DNA methylation can also lead to nucleolar stress, which is involved in the pathogenesis of aging and many related diseases [26].

## rDNA methylation and diseases

The consequences of impaired ribosomal biosynthesis resulting from rDNA methylation reach far beyond the scope of aging, extending into the intricate landscape of diseases. The resulting nucleolar stress, linked to the pathogenesis of aging, serves as a pivotal link connecting rDNA methylation to a multitude of diseases. This connection prompts an exploration into the specific nuances of how alterations in ribosomal DNA methylation may contribute to the onset and progression of various diseases, thereby unraveling a deeper understanding of the complex interplay between epigenetic modifications and pathological processes.

### rDNA methylation and neurological diseases

Decreased rRNA levels and reduced ribosomal activity have been linked to the pathological progression of Alzheimer’s disease (AD) [61–63]. Separate studies have indicated elevated rDNA methylation and increased rDNA content in the cerebral cortex and cerebellum regions of AD patients compared to age-matched controls [34, 64]. Similarly, increased rDNA methylation has been observed in the cortical region of patients with mild cognitive impairment (MCI), an early stage of AD. In the early stages of AD, there is an increase in rDNA methylation, whereas in the late stages, there is an enrichment of low-methylated rDNA units. In contrast, patients with MCI exhibit a broader range of global methylation, with higher methylation levels compared to late-stage AD patients [34]. For another neurological disease, autism spectrum disorder (ASD), a comparative analysis involving 16 ASD samples and 11 control samples did not reveal any significant alterations in rDNA copy number or DNA methylation in the brains of individuals with ASD [35]. Similarly, using 53 neuronal and 42 oligodendrocyte samples, no changes in rDNA methylation or copy number associated with schizophrenia (SCZ) were observed [35].

### rDNA methylation and hematologic diseases

With the increase of age, the activity and function of the hematopoietic system decline, which is manifested by the decrease of the number of lymphocytes and the weakening of hematopoietic stem cells (HSC) differentiation function. In CD34 + cells from myelodysplastic syndrome (MDS) patients, there is increased methylation of rDNA promoters, leading to reduced expression of rRNA and disruption of ribosomal biogenesis compared to healthy controls. It is also explored the hypothesis that hypomethylating agents induced an increase in intracellular rRNA transcription, thereby reducing apoptosis, and obtained support from recent data [36].

### rDNA methylation and genetic disorders

Werner’s syndrome (WS), also known as progeria short stature disease or adult progeria syndrome, is characterized by premature aging of the skin, a senile appearance, short stature, degeneration of the cardiovascular system, and a higher risk of diabetes and malignant cancer. In vitro studies examining rDNA in fibroblasts from both young and old normal individuals, as well as WS patients, revealed significant hypermethylation in samples from normal elderly individuals and WS patients. This hypermethylation leads to decreased expression of RNA pol II-transcribed genes. Moreover, the life span of fibroblasts in WS patients is significantly shortened [37].

Down syndrome (DS), also known as trisomy 21, is the most common viable chromosomal aneuploidy. The characteristic features of this condition include cognitive impairments, craniofacial anomalies, and early infantile hypotonia [65]. In a study utilizing targeted deep sequencing to assess DNA methylation in the rDNA of 69 adult individuals with DS and 95 controls using whole blood samples, elevated methylation level were identified in the promoter region of rDNA in individuals with DS compared to controls [38]. It has been reported that a significant positive correlation between 45S rDNA methylation and relative rDNA copy number in multiple tissues [35]. Trisomy of chromosome 21 results in most Down syndrome patients having an additional 30–40 copies of the rDNA unit. The increased rDNA methylation leads to silencing of these additional rDNA copies. It may be a dosage compensation mechanism, which ensures the homoeostatic regulation of ribosome biogenesis.

### rDNA methylation and cancers

#### rDNA methylation and sex hormone-regulated cancers

Breast cancer, ovarian cancer, cervical cancer and endometrial cancer are among the major malignancies that adversely affect women’s health worldwide. As early as 2000, it was reported that the average percentage of rDNA methylation in breast tumors was notably higher compared to normal control samples. This increase was associated with the inhibition of estrogen receptor expression [39]. In 2014, further validation of this phenomenon revealed increased methylation at the promoter, 18S, and 28S regions of rDNA. Additionally, DNA methylation levels of several CpG sites within the rDNA locus were found to correlate with the nuclear grade and nucleolar size of tumor tissues [40]. Research on ovarian cancer tumor samples indicated significantly higher methylation levels of 18S and 28S rDNA compared to normal ovarian surface epithelial samples. Furthermore, this hypermethylation could predict the progression-free survival of ovarian cancer patients. Treatment with the demethylating drug 5’-aza-2’-deoxycytidine showed that the rDNA hypermethylation in ovarian cancer led to gene silencing [41]. Investigation into the role of rDNA methylation status and rRNA expression level in cervical intraepithelial neoplasia (CIN) specimens revealed rRNA overexpression in cervical cancer cells, accompanied by demethylation of rDNA promoter and decondensation of rDNA, presumably promoting protein synthesis to support cancer cell proliferation and expansion. Although reports on the multistage development of the disease are still lacking, this study suggested that demethylation of rDNA promoter in cervical cancer cells leads to increased rRNA expression [42]. Analysis of rDNA methylation in 215 endometrial tumors showed that the majority of the tumors exhibited elevated levels of rDNA methylation. Patients with lower levels of rDNA methylation demonstrated significantly worse disease-free survival and overall survival [43].

Prostate cancer ranks as the second most prevalent malignancy among men globally, trailing only lung cancer [66]. Research conducted on human prostate cancer cell lines and clinical samples unveiled no significant correlation between rDNA promoter methylation and rRNA overexpression. Furthermore, it indicated that MYC governs rRNA levels in human prostate cancer, implying a molecular mechanism potentially contributing to nucleolar alterations [44]. However, a recent study suggests that prostate cancer demonstrates reduced methylation in the IGS region of rDNA and heightened variability of rDNA compared to normal tissues [45].

#### rDNA methylation and other types of cancers

In addition to sex hormone-regulated cancers, variability in rDNA methylation has also been noted in oral squamous cell carcinoma, esophageal cancer, hepatocellular carcinoma, lung cancer, and colorectal cancer. In oral squamous cell carcinoma, rRNA expression is suppressed compared to normal tissues, but no significant DNA methylation changes were observed in the rDNA promoter region [46]. Representative CpG sites of rDNA exhibit a significant decrease in average methylation levels and an increase in variability in esophageal cancer and hepatocellular carcinoma. Partial tumor samples from colorectal cancer, liver cancer, and lung cancer also displayed hypomethylation. Furthermore, the methylation profile of rDNA in cell-free DNA from plasma can serve as a biomarker for the detection of various cancers, including colorectal cancer, lung cancer, and liver cancer [45].

To summarize, dysregulated rDNA methylation is a common feature observed in a range of diseases, including neurodegenerative diseases, hematologic diseases, genetic disorders, and cancers. In ovarian and endometrial cancers, rDNA methylation levels can predict progression-free survival, while rDNA methylation in cell-free DNA from plasma can serve as a biomarker for detecting multiple cancers. Given the extensive remodeling of the epigenome in diseases, changes in rDNA methylation are perhaps unsurprising. However, the key lies in identifying specific rDNA methylation changes that truly indicate disease progression and significantly impact disease development. This will provide valuable insights into disease mechanisms, facilitate the search for early diagnostic markers for diseases, and support therapeutic interventions.

## Conclusions

This review provides a comprehensive overview of advancements in research on rDNA methylation concerning embryonic development, age and diseases associations across multiple species. During the preparatory stages of embryonic development, germ cells exhibit lower rDNA methylation compared to somatic cells. Following embryo implantation, there is a remethylation of the rDNA promoter, leading to overall higher methylation levels within the embryo than outside. As organisms age, rDNA methylation increases in the sperm and oocyte of humans, rats, and mice. Various tissues across species, including liver, heart, bone marrow, and skeletal muscle, display heightened rDNA promoter methylation. Changes in rDNA methylation levels are also observed in various types of diseases. rDNA methylation serves not only as an epigenetic clock across multiple species but also as a biomarker for the detection and prognosis of various diseases.

The genetic identity of rDNA units plays a crucial role in determining their epigenetic state. Genetic variations within the rDNA sequence can lead to the emergence of different epialleles, which in turn affect rDNA methylation patterns [67]. For example, specific genetic variants may predispose rDNA units to increased methylation, serving as a mechanism to silence additional rDNA copies. During development, aging, and disease, alterations in rDNA methylation may be influenced by genetic variations associated with rDNA [67], and concurrently coordinated with changes in rDNA copy number [35], thereby potentially regulating rDNA transcription, resulting in an overall modification in protein synthesis rates in the organism. Dysregulation of cellular protein synthesis may significantly contribute to the gradual deterioration of cellular functions associated with aging and diseases.

Despite these advancements, there remains a noticeable gap in research concerning the dynamic patterns of rDNA methylation throughout the entire embryonic development process across various species, as well as its systemic evolution. Questions persist regarding whether rDNA methylation influences processes such as cell differentiation, orientation, and proliferation. Additionally, the precise causal relationship between rDNA methylation and disease or aging process remains elusive. One of the challenges hindering rDNA research is that complete rDNA sequences are typically absent from most standard genome assemblies. The highly repetitive nature of rDNA makes it difficult to accurately analyze and locate rDNA within genome sequences [16, 68]. Additionally, rDNA copy number variation and the characteristic dense tangles further complicate the accurate assignment of reads from sequencing data, as well as the precise identification and quantification of these regions [69].

To advance our comprehension of rDNA methylation, researchers have focused on assembling the rDNA sequence and structure in the human genome. They have employed a specific method to separate the rDNA methylated region from whole-genome bisulfite sequencing (WGBS) data [45]. Additionally, by utilizing the Arachne assembler method, researchers have enhanced the suitability of the reference genome for rDNA localization, enabling precise insertion of rDNA tandem repeat sequences [70]. Recently, researchers have also customized five human and mouse reference genome assemblies, incorporating known rDNA sequences, creating annotation files, and proposing a straightforward workflow for researchers to map and visualize rDNA sequences more effectively [68]. Despite these advancements, the method for assembling rDNA sequences has not been widely embraced, resulting in a dearth of database resources displaying rDNA methylation information. Hence, there are plans to incorporate rDNA methylation profiles from various biological states, such as development, aging, and diseases, into our independently developed MethBank database, which contains cross-species single-base resolution DNA methylation data [21]. By identifying more stable and reliable rDNA methylation markers associated with aging, diseases, and development across multiple species, this endeavor aims to provide new directions for disease detection and intervention based on rDNA methylation, as well as clues for understanding the mechanisms underlying rDNA methylation’s involvement in diverse biological processes.

## Data Availability

No datasets were generated or analysed during the current study.

## Abbreviations

AD: Alzheimer’s disease
ASD: Autism spectrum disorders
CIN: Cervical intraepithelial neoplasia
CPE: Core promoter elements
DNMT: DNA methyltransferase
DS: Down syndrome
E4.5: Embryonic day 4.5
E5: Embryonic day 5
E7.5: Embryonic day 7.5
E13.5: Embryonic day 13.5
E18.5: Embryonic day 18.5
HSC: Hematopoietic stem cells
IGS: Intergenic spacer
MCI: Mild cognitive impairment
MDS: Myelodysplastic syndrome
NORs: Nucleolar organizer regions
NuRD: Nucleosome remodeling and histone deacetylase
OSCC: Oral squamous cell carcinoma
rDNA: Ribosomal DNA
RFB: Replication fork barrier
SCZ: Schizophrenia
TTF-1: Transcription termination factor-1
UCE: Upstream control elements
WS: Werner’s syndrome
WGBS: Whole-genome bisulfite sequencing
